# The osteopath-parent-child triad in osteopathic care in the first 2 years of life: a qualitative study

**DOI:** 10.3389/fpsyg.2023.1253355

**Published:** 2023-10-02

**Authors:** Caterina Accardi, Francesco Cerritelli, Lorenza Bovo, Jorge E. Esteves

**Affiliations:** ^1^Foundation COME Collaboration, Clinical-Based Human Research Department, Pescara, Italy; ^2^Malta ICOM Educational Ltd., Gzira, Malta

**Keywords:** therapeutic alliance, active inference, affective touch, synchrony, predictive processing, enactivism, osteopathy, perinatal care

## Abstract

**Background:**

Enactivism and active inference are two important concepts in the field of osteopathy. While enactivism emphasizes the role of the body and the environment in shaping our experiences and understanding of the world, active inference emphasizes the role of action and perception in shaping our experiences and understanding of the world. Together, these frameworks provide a unique perspective on the practice of osteopathy, and how it can be used to facilitate positive change in patients. Since the neonatal period is a crucial time for development, osteopaths should aim to create a therapeutic relationship. Arguably, through participatory sense-making, osteopaths can help the baby build a generative model (with positive priors) to deal with stress and needs throughout their life.

**Aim:**

Since the literature considers that interactions with the environment, which enact the patients’ experiences, depending on contextual factors and communication between patient and caregiver, this research explored whether there is a correspondence between the indications in the literature and clinical practice in the management of the mother/parent–child dyad during osteopathic care on children aged 0 to 2 years old.

**Methods:**

Semi-structured interviews were conducted with a purposive sample of nine osteopaths with experience in the field of pediatrics. Interviews were transcribed verbatim, and constructivist grounded theory was used to conceptualize, collect and analyze data. Codes and categories were actively constructed through an interpretive/constructionist paradigm.

**Results:**

The core category was the idea of the pediatric osteopath as a support for the family, not only for the child. Four additional categories were identified: (1) Preparing a safe environment for both children and parents, (2) Communication, (3) Attachment and synchrony, and (4) Synchronization.

**Conclusion:**

Through participatory sense-making, osteopaths manage contextual factors to establish an effective therapeutic alliance through the osteopath-parent–child triad to facilitate the construction of the child’s internal generative model to promote healthy development. The therapeutic encounter is considered an encounter between embodied subjects, occurring within a field of affordances (ecological niche) that allows the interlocutors to actively participate in creating new meanings through interpersonal synchronization. Participatory sense-making and the establishment of a therapeutic alliance through the osteopath-parent–child triad are crucial to promote healthy development in the child.

## 1. Introduction

Enactivism challenges the conventional division of mind and world, emphasizing the interdependent nature of organisms and their environment. As individuals navigate through life, their interactions with other people shape their perceptual experiences, which are constructed based on prior beliefs or “priors” that form internal generative models and minimize prediction errors (Gallagher, 2005; Sørvoll et al., 2022). Sense-making, defined as an autonomous system’s ability to adaptively regulate its operations and establish a mutually influential relationship with the environment, plays a vital role in maintaining viability as a form of life (Di Paolo, 2020). Being a sense-maker entails selectively acting on the affordances offered by the environment, crucial for understanding the sense of agency (Segundo-Ortin, 2020).

Osteopathy is a person-centered healthcare discipline that recognizes the intricate interplay between the body’s structure, function, and self-regulatory mechanisms. It takes a comprehensive approach to health and well-being, considering the entirety of the individual (European Standards EN 16686, 2015). It is therefore important to go beyond perceiving osteopathy solely as a body-centered intervention based on aetiological models of care (Esteves et al., 2020). Human functioning is complex, unique to each individual, and unpredictable. In this regard, embracing the ecological niche becomes crucial. Within the patient-practitioner dyad, a multitude of affordances are provided, allowing for adaptations and the restoration of productive agency. This process is facilitated by the active engagement of both parties involved (Ramstead et al., 2019).

Enactive theory interprets organisms as co-emerging and co-determining entities within their (social) environment. Even at the earliest stages of development, basic perception and action emerge, fostering active, bidirectional co-regulation referred to as “co-embodiment” and “co-homeostasis” between the developing fetus and its surrounding womb (Zieber et al., 2015; Ciaunica et al., 2021a).

The fetus interacts with its environment, adapting its generative model based on the mother’s own (Ciaunica et al., 2021b). This intriguing interplay underscores the dynamic nature of fetal development. Moreover, biological self-organization in humans encompasses a remarkable array of processes, ranging from developmental milestones to metabolic intricacies, immunity mechanisms, and the orchestration of endocrine functions (Koban et al., 2021). Such intricate self-organization reflects the profound interconnectedness of our physiological systems.

In light of these insights, the maternal role emerges as a key influencer in shaping the developing child’s physiological landscape. Maternal behaviors trigger endocrine and metabolic responses in the unborn child, potentially contributing to the formation of “priors,” or anticipatory expectations, regarding the external world. Intriguingly, maternal engagement practices, such as tactile stimulation of infants’ bellies, might establish an environment conducive to heightened sensitivity to touch and tactile interactions in newborns (Ciaunica et al., 2023). This maternal influence on sensory predispositions could lay the groundwork for how infants perceive and respond to their surroundings.

Considering these intriguing foundations, a captivating question emerges: Could these “priors” regarding tactile stimuli also render newborns more receptive to hands-on osteopathic interventions? The notion of “priors” extends beyond the realm of sensory perception and has the potential to influence how infants process and respond to external stimuli, including therapeutic touch. By delving into the intricate interplay among prenatal maternal influences, sensory development, and the potential implications for osteopathic interventions, we could open the door to a captivating avenue of investigation.

As we delve into the multifaceted connections between maternal interactions, sensory sensitivities, and osteopathic interventions, it becomes evident that the journey of discovery holds promise for enhancing our understanding of both prenatal influences and postnatal therapeutic possibilities. The intricate web of interactions, as illuminated by recent research, encourages us to envision a holistic approach that acknowledges the prenatal origins of sensory propensities and their potential implications for osteopathic care.

Arguably, the interplay between maternal behaviors, fetal development, and sensory priors represents an exciting frontier of investigation. This nexus between prenatal influences and postnatal responses underscores the nuanced ways in which early experiences shape our perceptual world. By exploring these relationships, we not only deepen our appreciation for the complexities of human development but also lay the groundwork for innovative approaches to healthcare, such as osteopathic interventions.

Bowlby’s attachment theory elucidates the formation of attachment between an infant and their caregiver during the initial 2 years of life, with profound implications for long-term development (Zimmermann and Iwanski, 2019). Adverse prenatal and childhood environments are causally linked to the risk of chronic illness in adulthood (Mauder and Hunter, 2008). Attachment experiences and the expression of internal operating patterns contribute to illness behavior, somatization, and hypochondria. Infants often exhibit comorbidities associated with prematurity, birth complications, maternal stress, and perinatal stress (Tribe et al., 2018; Lobmaier et al., 2020). The early years are marked by high brain plasticity, which amplifies vulnerability to stressors but also presents an opportunity for resilience when appropriate support and interventions are in place. Recent research indicates that maternal care can epigenetically influence the offspring by inducing lasting changes in the hypothalamic–pituitary–adrenal axis, increasing the expression of glucocorticoid receptors in the hippocampus, and influencing sensitivity to bodily feedback during stressful situations (Meaney and Szyf, 2005; Mariani Wigley et al., 2021). Studies involving preterm infants in intensive care units reveal that maternal contact and breastfeeding contribute to neurological protection and proper brain development (Park and Kim, 2019).

The infant and toddler years constitute a critical period for the development of the child’s minimal self, feelings of security, self-esteem, and self-affirmation (Dieguez and Lopez, 2017). The concept of the minimal self arises from the dynamic integration of various sensory inputs from the body and the environment, including visual, tactile, proprioceptive, vestibular, auditory, olfactory, and visceral signals. Both spatial and social factors shape the interaction between the self and others’ bodies (Brugger and Lenggenhager, 2014; Dieguez and Lopez, 2017). The sense of ownership relates to the state of one’s body, while the sense of participation/action involves intention and the execution of actions. These interconnected perceptual systems contribute to the infant’s consciousness, forming an “embodied self” rooted in bodily experiences that emerges shortly after birth and continues to develop and function throughout childhood and beyond (Gallagher, 2005).

When examining the concept of human selfhood, the immediate and fundamental environment for a developing self-organizing human organism is another biological self-organizing system, specifically the mother’s body (Ciaunica et al., 2021b). It can be argued that the way in which the mother or father interact with the mother’s belly during pregnancy, or whether the mother receives osteopathic treatment, can influence the child’s experience of touch. Considering the available evidence on touch during early stages of development, which indicates its potential to attenuate brain activity evoked by noxious stimuli during painful procedures, decrease heart rate (HR), increase oxygen saturation (SpO2), promote respiratory sinus arrhythmia (an indicator of vagal tone), and reduce crying time (Manzotti et al., 2020), we can hypothesize that the type of touch experienced during the prenatal period may predict how touch is perceived after birth and the potential positive effects of osteopathic manipulative treatment.

The enactivist paradigm underscores an osteopath’s multifaceted role that extends beyond mere treatment to encompass active support and profound understanding of the patient’s unique illness experiences. This holistic approach involves the cultivation of fresh narratives that center on the patient’s altered physical capacities, evolving identity, their intricate connection with the internal and external milieu, and the acquisition of essential life skills (Esteves et al., 2020, 2022). In this light, therapeutic interventions are urged to align with a contemporary multidimensional model of care that interweaves insights from both neurophysiology and psychology, all while fostering active patient engagement (Esteves et al., 2022).

Clinically speaking, any healthcare intervention is an intricate fusion of two pivotal factors: (I) the intervention’s intrinsic biological components and (II) its surrounding contextual and psycho-social facets (Benedetti, 2013; Rossettini and Testa, 2018). The recognition of contextual factors has emerged as a significant discourse, characterizing elements that exist external to the treatment itself, such as the administered therapy (e.g., osteopathic care). These contextual factors wield the power to amplify therapeutic outcomes, particularly in the realm of managing musculoskeletal disorders (Palese et al., 2019).

Since the literature considers that interactions with the environment enact patients’ experiences, dependent on contextual factors and communication between the patient and caregiver, this research explores whether there is a correspondence between the literature’s indications and clinical practices in managing the mother/parent–child dyad during osteopathic care for children aged 0 to 2 years old.

## 2. Methods

This qualitative study, employing a “grounded theory” approach, delved into the realm of clinical practice with a keen focus on social processes, actions, and the dynamic interplay of interactions (Thong et al., 2007). By adopting an interpretive/constructivist paradigm, the study aimed to illuminate the contextual tapestry that envelops the subject matter and to intricately decipher the diverse perspectives encapsulated within the narratives of the interviewed osteopaths (Thomson et al., 2014). The choice of this research approach was meticulously considered, as it resonated as a more fitting avenue for this inquiry in comparison to alternative methodologies. This selection was predicated on our intention to garner a comprehensive understanding of the osteopaths’ viewpoints on the subject, thereby discerning whether there exists a harmonious alignment between the realm of research and the nuanced realm of clinical practice. Ethical approval was obtained from the Malta ICOM Educational Research Ethics Committee, ensuring ethical compliance throughout the study.

### 2.1. Sample selection and recruitment

Strategic sampling, also known as purposeful sampling, was employed to ensure a targeted and comprehensive understanding of the phenomenon under analysis (Lunghi et al., 2021). The sample size was determined based on data saturation, where no new information emerged from the interviews (Thomson et al., 2014). Additionally, the concept of “information power” was considered, taking into account factors such as the study’s aim, sample specificity, established theory, quality of dialog during interviews, and analysis strategy (Malterud et al., 2016).

Ten experienced osteopaths were contacted via email, providing detailed information about the study’s purpose and methodology, as well as the participants’ role, and inviting their interest in participating in the interviews. They were contacted by one of the researchers (not the interviewer) who maintains a professional relationship with some of them. Informed consent, including permission for the use of personal data and audio recording of the interviews, was obtained and signed by all participants before the interviews. Nine osteopaths were enrolled in the study, with a balanced gender distribution of five men and four women, encompassing a diverse range of nationalities: specifically, two Canadians, one German, one Spanish, and five Italians. The participants’ ages exhibited a mean value of 47.5 years, with a standard deviation of 10.7. Furthermore, the average number of years since their graduation was 17.5 years, with a standard deviation of 8.8 years. The interviews were conducted in English with the first four participants and in Italian with the remaining five.

### 2.2. Inclusion and exclusion criteria

The recruitment process involved selecting experienced pediatric osteopaths who had been practising for a minimum of 5 years and held a master’s or postgraduate qualification in pediatrics, specializing in working with children aged 0 to 2 years. Exclusion criteria comprised pediatric osteopaths with less than 5 years of experience, lacking a master’s degree in pediatrics, or not having completed a postgraduate course in pediatrics and not English or Italian speakers (Thomson et al., 2014). Additionally, participants who possessed a personal relationship—neither a friendship nor a work relationship—with the first author, who conducted the interviews, were also excluded from the study.

### 2.3. Data collection

Data collection involved conducting semi-structured interviews that incorporated elements of grounded theory. The researcher endeavored to establish a purposeful and welcoming environment, maintaining a neutral and nonjudgmental stance to foster participants’ freedom in expressing their perspectives on the topic at hand (Lunghi et al., 2021). The interviews were designed to facilitate inductive, in-depth, discursive, and profound conversations with the participants.

**Figure 1:**
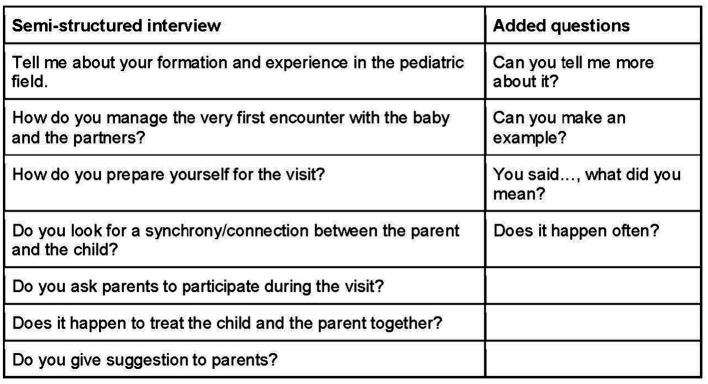
Interview questions.

To ensure the appropriateness and effectiveness of the interview questions and prompts, a rigorous process was followed. Two researchers with expertise in osteopathic clinical practice and research methodology reviewed the initial interview draft. Throughout the project, modifications were made to refine and adapt the questions based on the emerging themes raised by the interviewees (Figure 1).

Interviews were carried out via either FaceTime or telephone calls, enabling remote communication, and were conducted in either Italian or English. The primary researcher took detailed notes on participants’ comments during the interviews to aid in subsequent category formation and maintain adherence to the data. Additionally, each interview was recorded to facilitate transcription and data analysis. To ensure credibility, participants were given the opportunity to review their transcripts for accuracy and provide comments (member checking).

The interviews were scheduled with participants, with each session lasting between 30 and 60 min. Prior to each interview, participants were provided with an informed consent form that clearly outlined the study’s characteristics and the use of personal data. Throughout the study, participants’ anonymity was safeguarded and upheld. The transcripts of the interviews can be viewed on the Open Science “The osteopath-parent-child triad in osteopathic care”.

### 2.4. Quality criteria

The methodology and research process of the study were evaluated for quality using the criteria established by Thomson et al. (2014) and the Consolidated Criteria for Reporting Qualitative Research (COREQ) checklist proposed by Thong et al. (2007). To ensure trustworthiness in the study, a set of quality criteria was employed, which included elements of credibility, transferability, dependability, and confirmability (Vaismoradi et al., 2013; Cerritelli and Lanaro, 2018). The study was conducted in accordance with the ethical principles outlined in the Declaration of Helsinki (World Medical Association, 2013).

### 2.5. Data analysis

To ensure the transferability of the data, a comprehensive analysis process incorporating both inductive and deductive approaches was employed to create a thematic map that captured the context and integrated both manifest and latent contents of the data (Vaismoradi et al., 2013).

**Figure 2:**
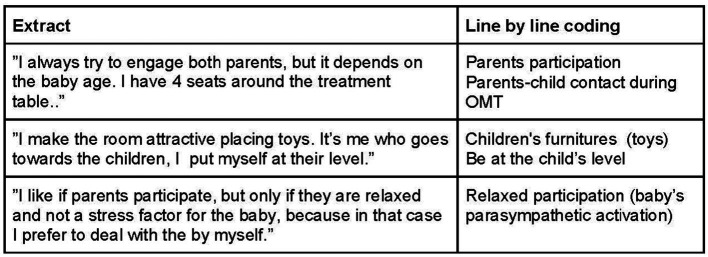
Line-by-line coding.

Memo-writing (Figure 2) was utilized as a tool by the researchers to compare data, facilitating initial analytical understanding and later theoretical exploration, as well as to examine the relationships between categories and concepts. These memos also served as reflective tools (Thomson et al., 2014). Constant comparison of the data enabled the identification of similarities and differences (Lunghi et al., 2021). Initial coding was conducted by analyzing the data line by line, followed by focused coding to evaluate the most significant codes (Thomson et al., 2014). Subsequently, a “central category” was selected to encompass and explain the entire grounded theory (Thomson et al., 2014). The process continued with ongoing comparative analysis of data and categories until “theoretical saturation” was reached (Thomson et al., 2014).

To enhance transferability and ensure reliability of the obtained data, two interviews were reanalyzed by another qualitative researcher, in addition to the first author reanalyzing two interviews. Additionally, two participants were re-interviewed at the end of all nine interviews to gain a deeper understanding and clarification of their responses. Furthermore, an audit logbook, referred to as verification trials or audit trial, was maintained to ensure dependability and confirmability by documenting the research process, data collection, and theory generation.

## 3. Results

The nine osteopaths who participated in the research will be identified by numbering from N1 to N9 to ensure their privacy. Data saturation was reached after the first seven interviews. Interviews with N8 and N9 were used to confirm data saturation, and two re-interviews were conducted with N2 and N3 to enhance data reliability.

### 3.1. Categories

**Figure 3:**
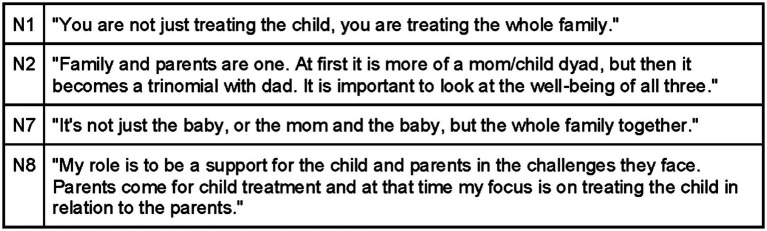
Core categories.

The core concept that encompasses the entire theory put forth in this research is the participants’ perception of themselves as not only clinicians treating the baby but also as clinicians providing support to the entire family (Figure 3). From the interviews, four main themes or categories emerged, each consisting of sub-themes or subcategories that form the foundation of the theory proposed in this research. These four categories represent sequential steps in a hypothetical model for conducting a pediatric visit in osteopathy, with a particular emphasis on the contextual factors influencing the treatment process:

1. Creating a safe environment for children and parents.
2. Communicating.
3. Looking for attachment and synchrony.
4. Synchronizing.

### 3.2. Theme 1: creating a safe environment for children and parents

**Figure 4:**
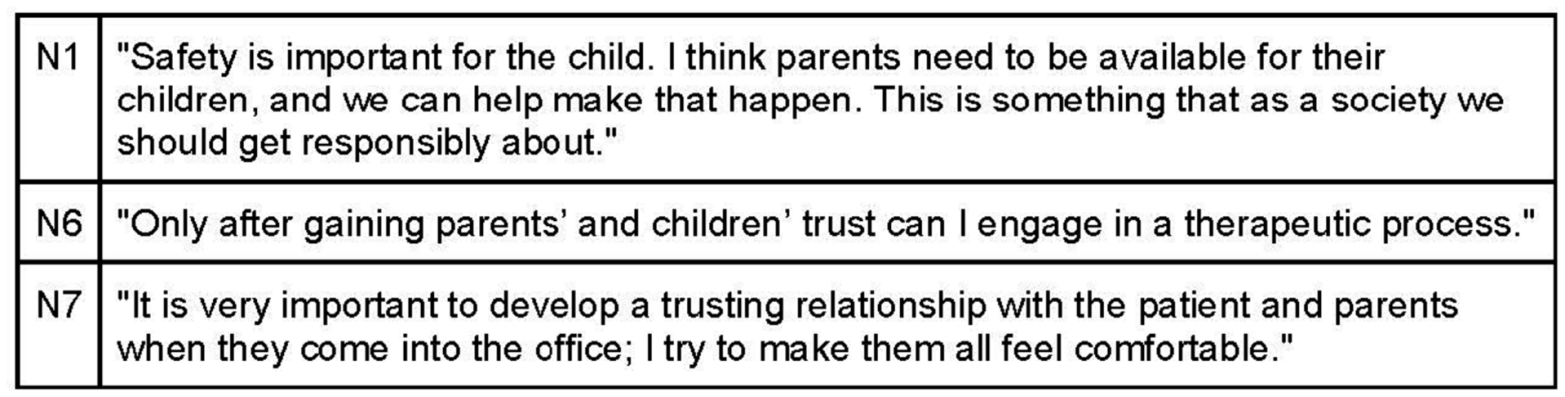
Safety environment by developing a trusting relationship.

**Figure 5:**
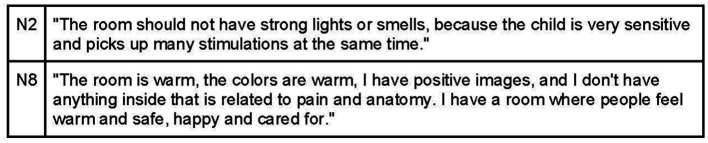
Positive room setting.

**Figure 6:**
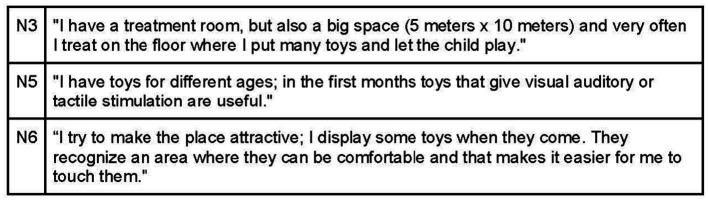
Room setting for child’s engagement.

The osteopathic visit is a therapeutic interaction that occurs within a designated space, influenced by the room and the osteopath. According to the interviews, the practitioner’s objective is to create a welcoming environment where participants feel comfortable expressing themselves, reassured, and free from judgment (Figure 4). Interviewees commonly believe that the office should exude warmth and hospitality, achieved through elements such as warm colors, soft lighting, and soothing sounds. It is important to avoid overstimulating children to prevent irritation (Figure 5). Participants also mentioned the use of toys on the floor to engage children as active participants in the environment (Figure 6).

**Figure 7:**
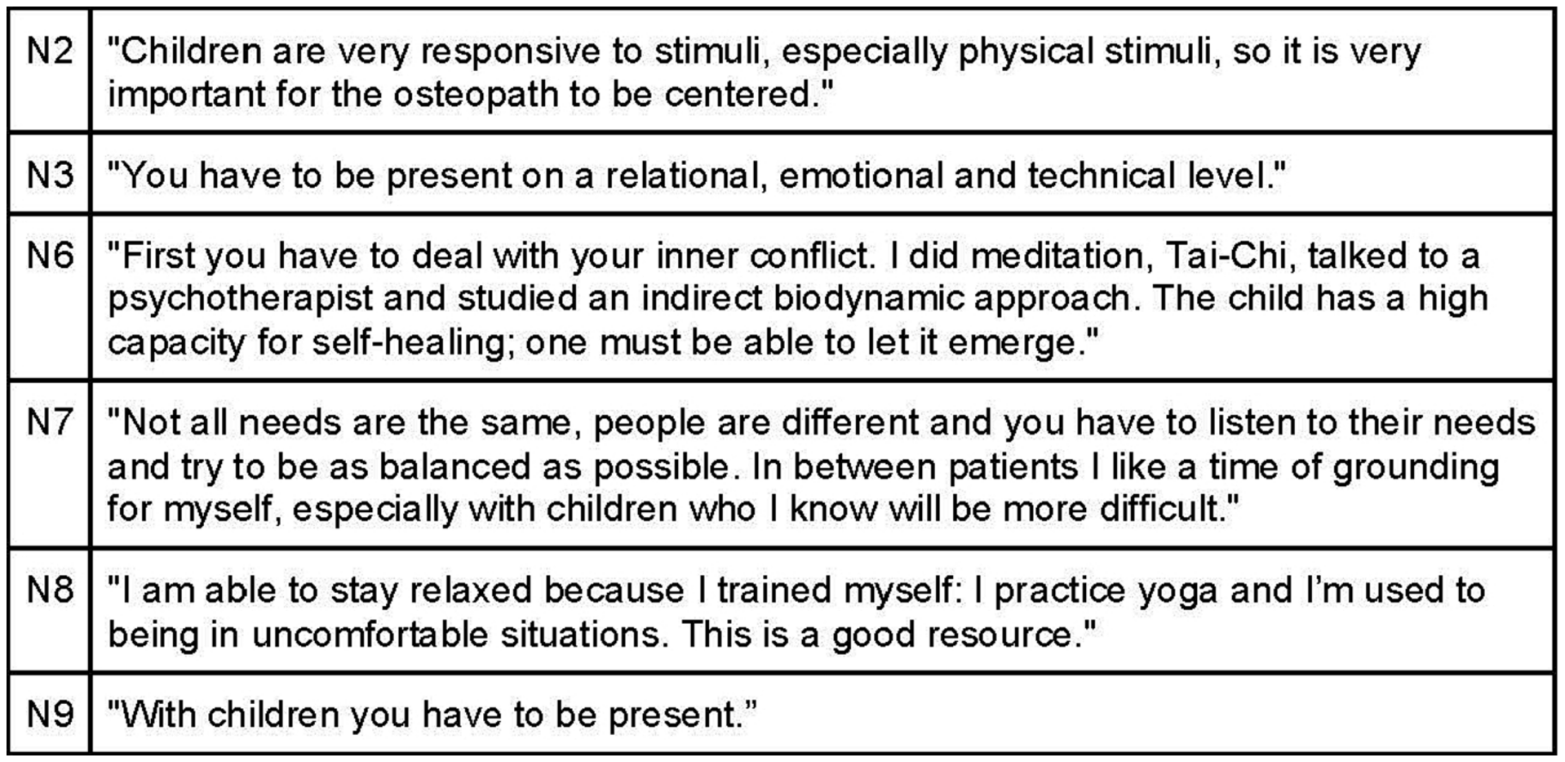
Osteopath’s self-awareness.

**Figure 8:**
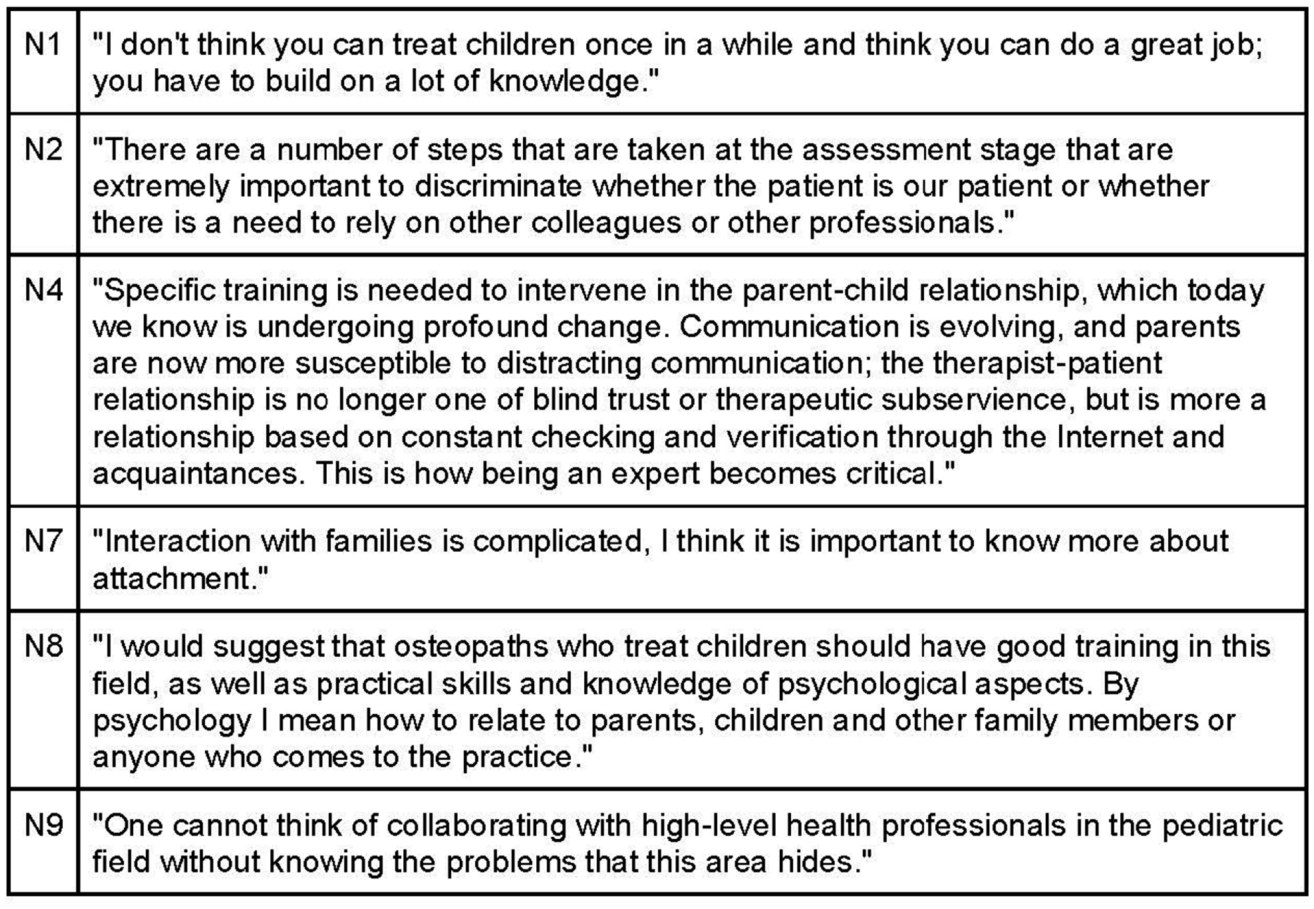
Osteopath’s knowledge.

Osteopaths place great emphasis on self-awareness to establish a connection with their patients. Many engage in practices like meditation, undergo psychotherapeutic training, and develop extensive knowledge and skills in pediatrics and communication (Figure 7). Building trust with parents is facilitated by demonstrating up-to-date scientific knowledge and confidence in their expertise. Being a pediatric osteopath requires not only manual dexterity but also a strong understanding of pathophysiology and in-depth knowledge of neurobehavioral development (Figure 8).

### 3.3. Theme 2: communicating

**Figure 9:**
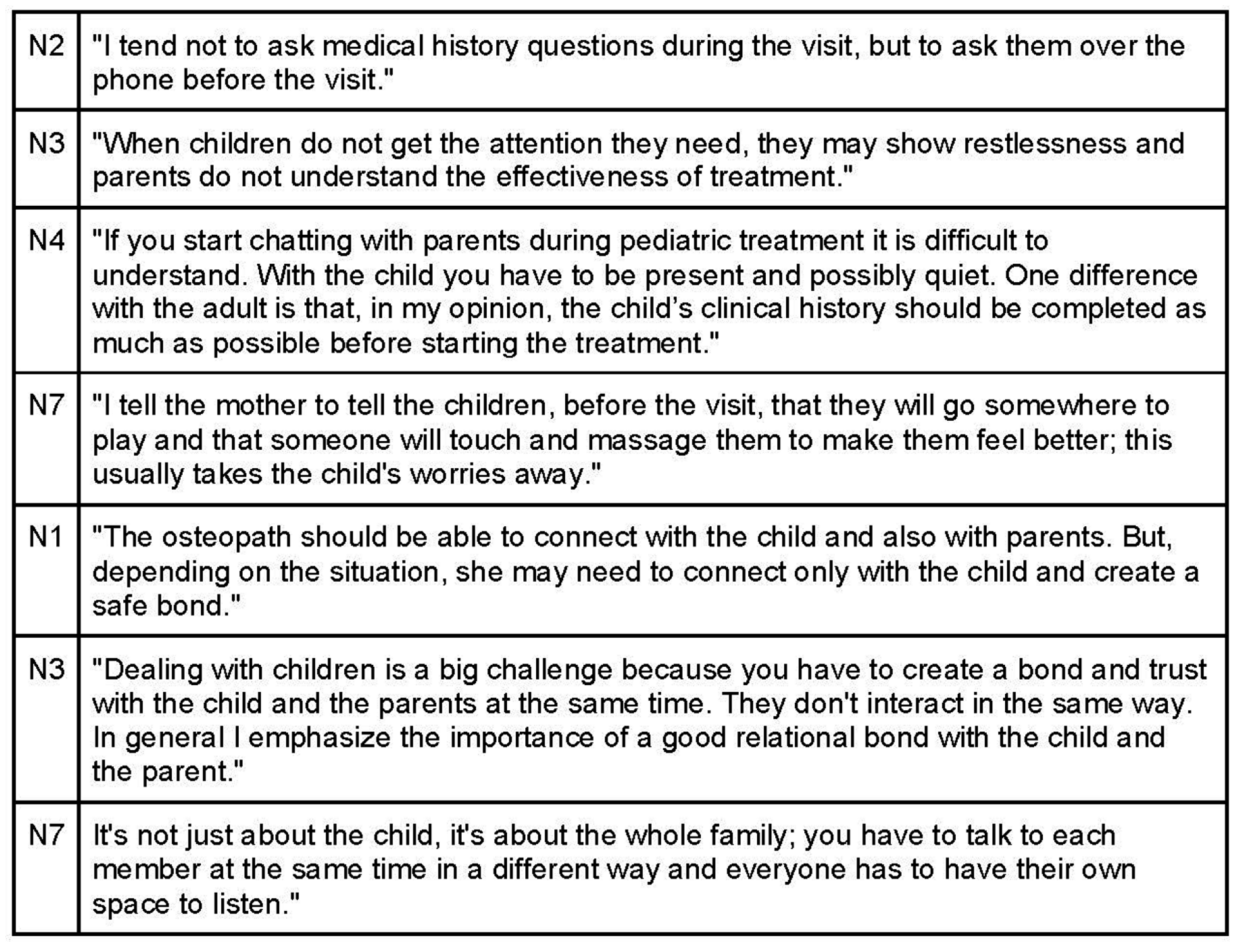
How to build a therapeutic alliance.

**Figure 10:**
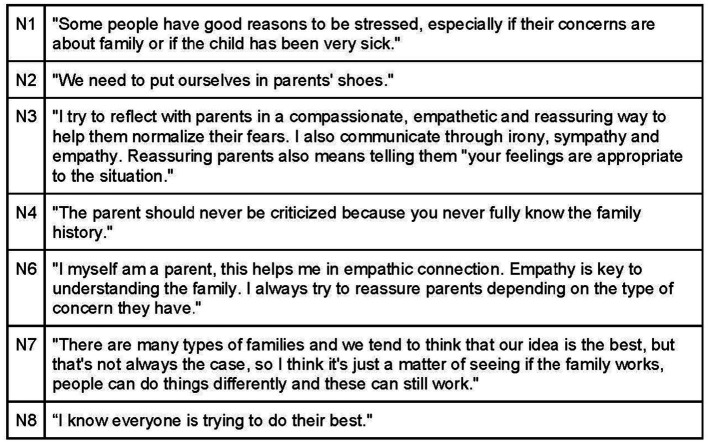
Therapeutic alliance through effective communication with parents.

**Figure 11:**
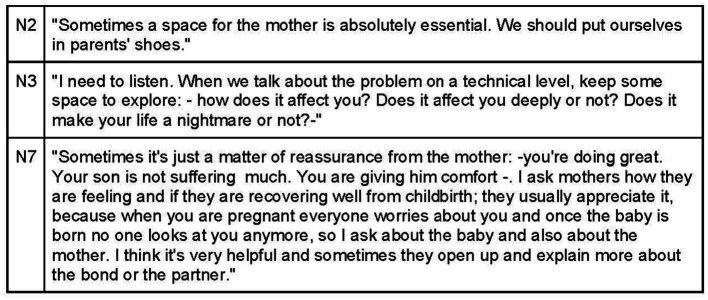
Therapeutic alliance between mothers/caregivers and the osteopath.

**Figure 12:**
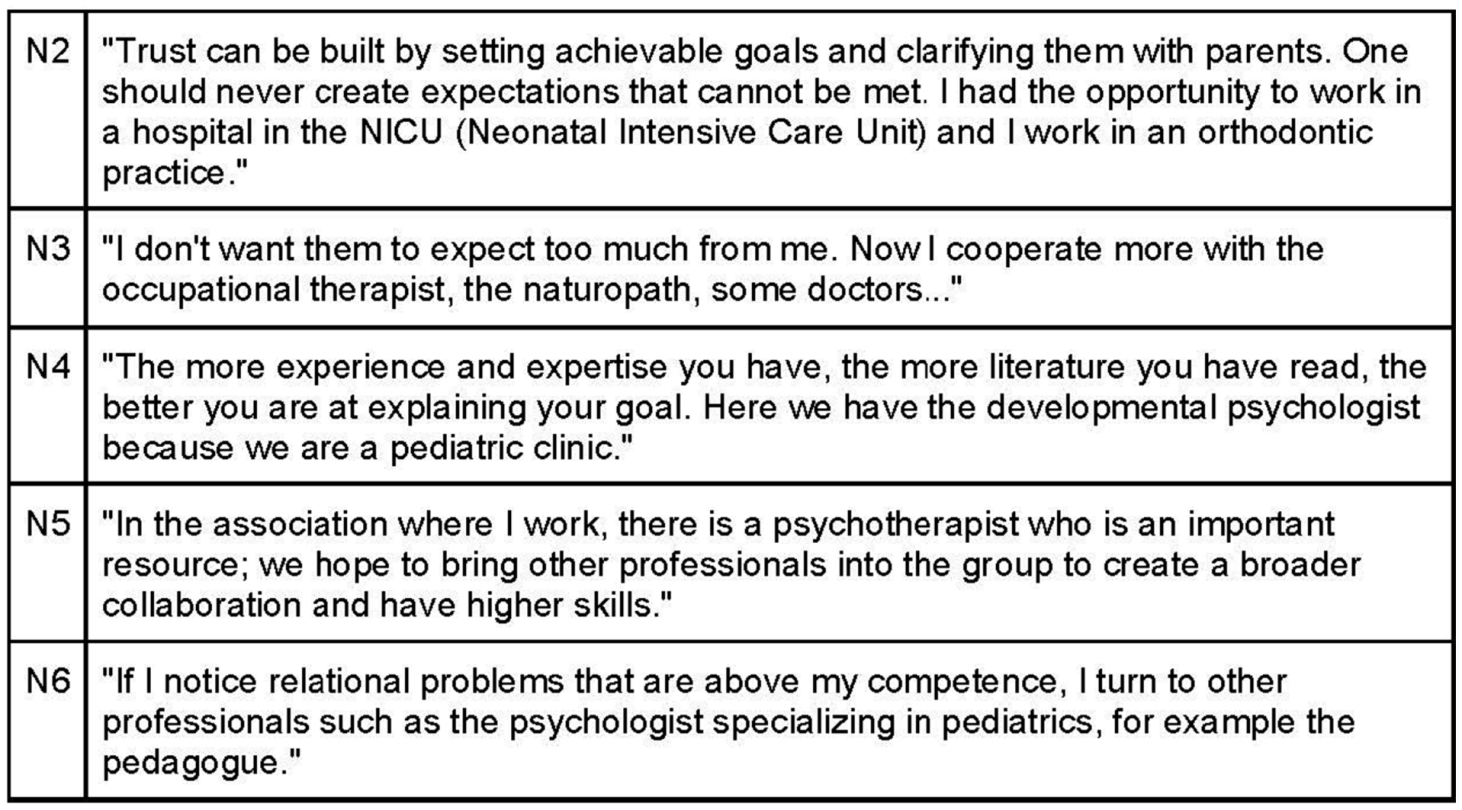
Therapeutic alliance sharing goals and working in a team.

Investing time in creating a therapeutic alliance based on empathy and collaboration is essential, with acknowledgement of each participant in the therapeutic encounter (Figure 9). Effective communication with parents entails demonstrating empathy, calmness, and a desire to understand their feelings and experiences with their child (Figures 10, 11). Sharing achievable goals and collaborating with other professionals, such as pediatricians and midwives, contributes to a strong therapeutic alliance (Figure 12).

**Figure 13:**
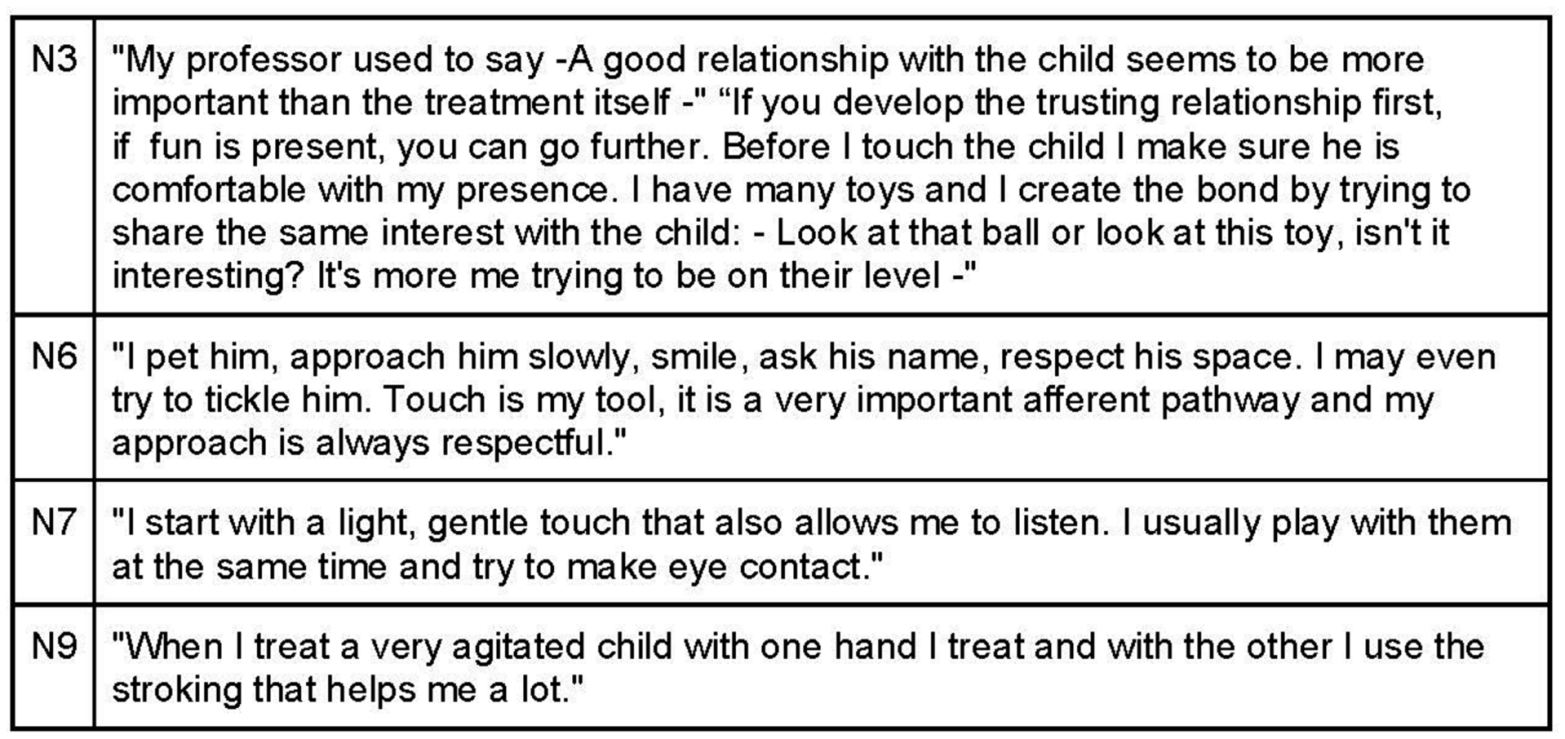
Therapeutic alliance between the child and the osteopath.

Establishing a rapport with the child involves engaging with them on an equal level, often by initiating interaction on the floor through play or approaching them while in their parents’ arms. Eye contact is highlighted as a crucial form of communication, considering the child’s abilities and development. The osteopath should initiate contact with a gentle touch, starting from less sensitive areas and gradually moving toward more central and sensitive regions (Figure 13).

### 3.4. Theme 3: looking for attachment and synchrony

**Figure 14:**
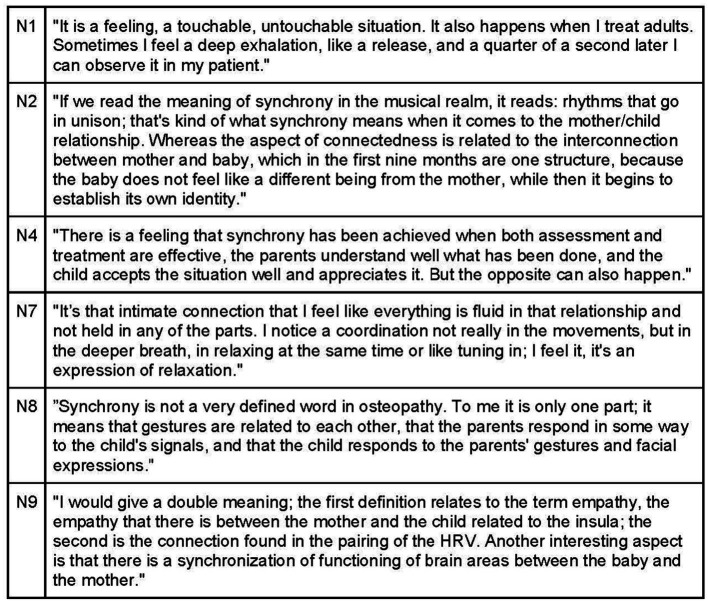
Synchrony.

**Figure 15:**
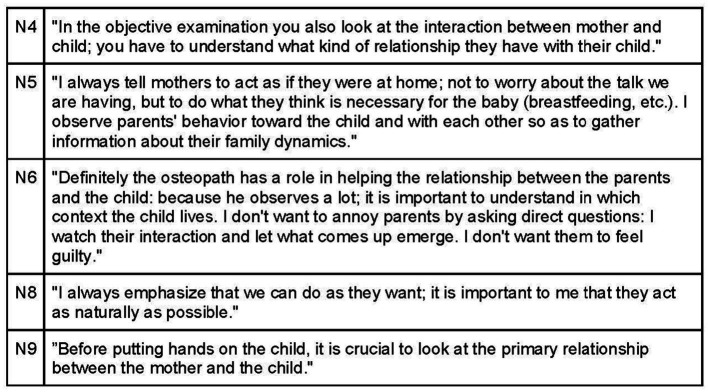
Synchrony between child and their caregivers.

Participants universally recognize the significance of the parent–child connection and synchronicity, as it provides insights into the attachment pattern and its impact on both parties (Figure 14). During the visit, natural behavior from parents and the child is encouraged, as it facilitates the observation of attachment and interaction dynamics that may contribute to the child’s symptoms. Noticing any mismatched rhythms, hurried approaches, or signs of stress or agitation in the parent’s handling of the child is crucial (Figure 15).

### 3.5. Theme 4: synchronizing

**Figure 16:**
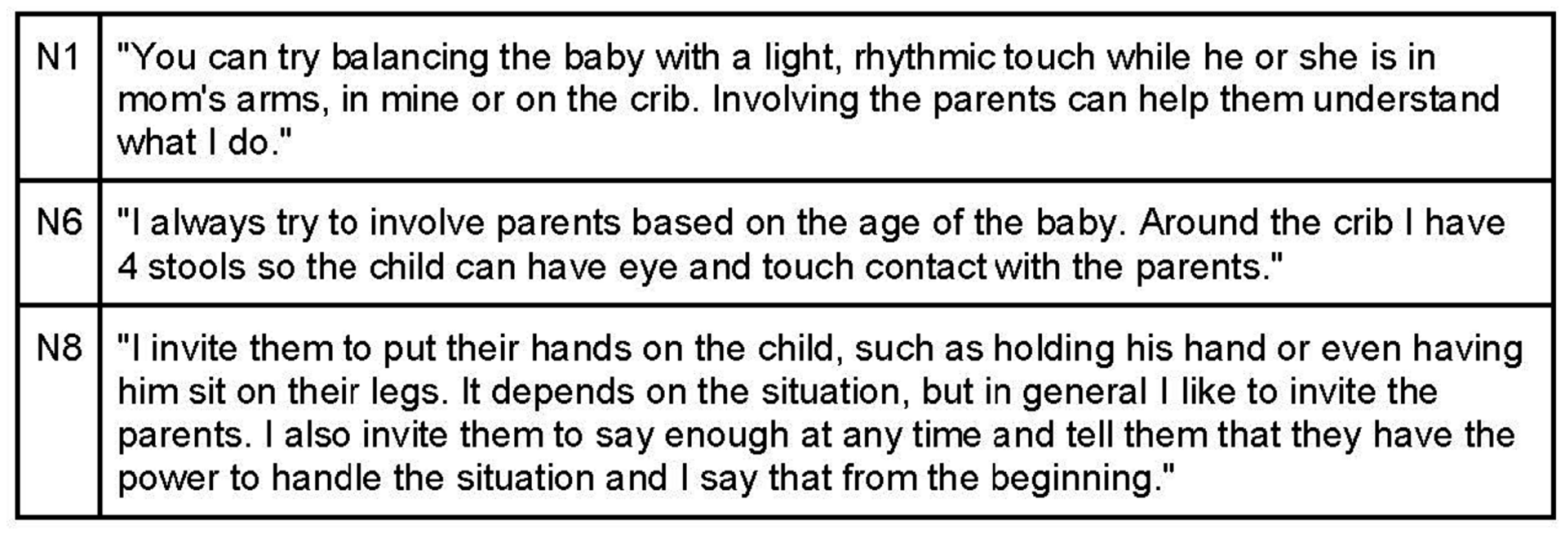
Caregivers involvement during osteopathic treatment sessions.

Participants value involving parents in the visit, encouraging them to sit around the treatment table to maintain eye and touch contact with the baby. Parental involvement can include gentle touching, movement, or nursing, always in a relaxed manner. If parent participation causes concern for the baby, osteopaths prefer to handle the baby themselves (Figure 16).

**Figure 17:**
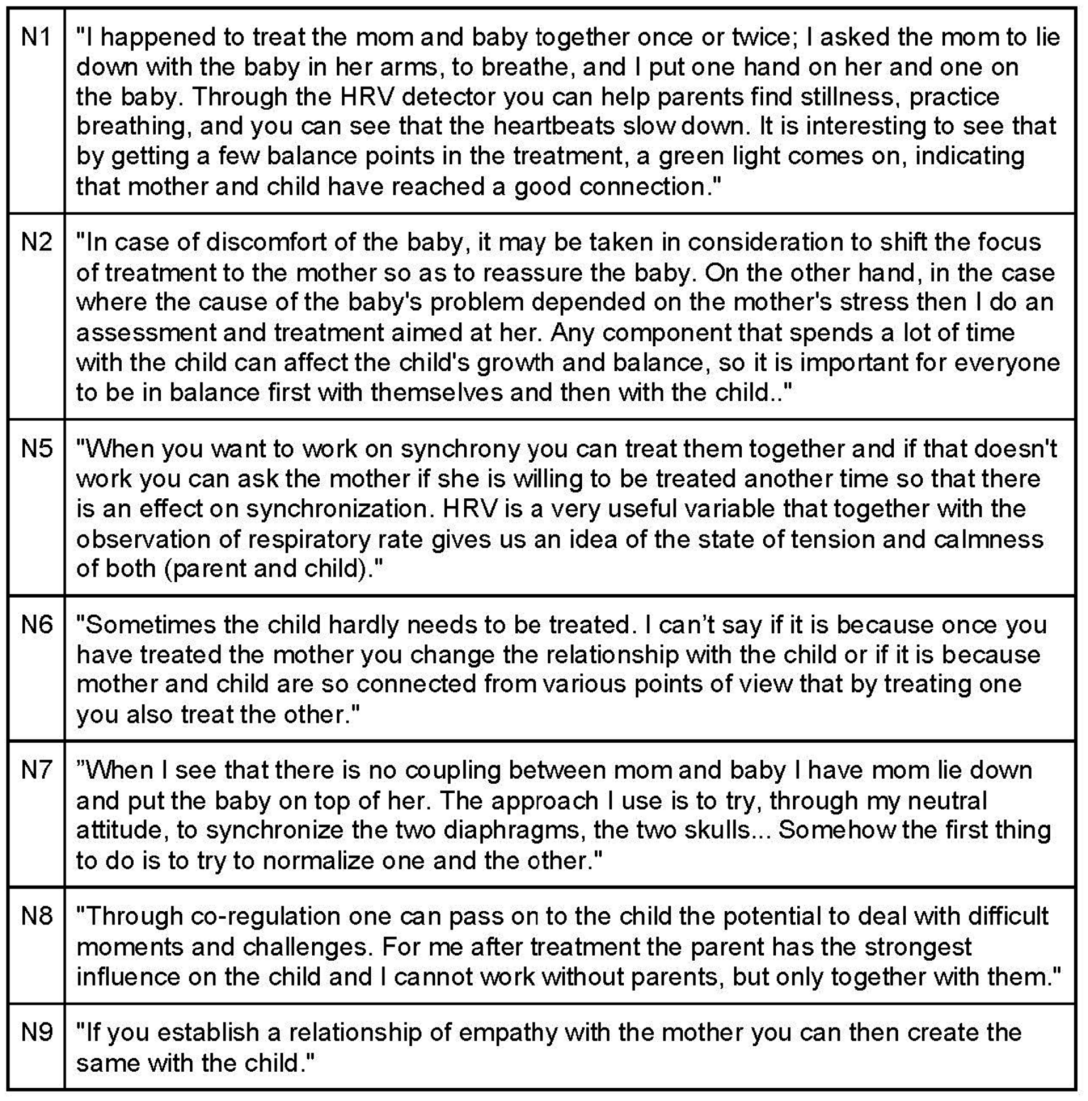
Caregiver-child-osteopath synchronization.

**Figure 18:**
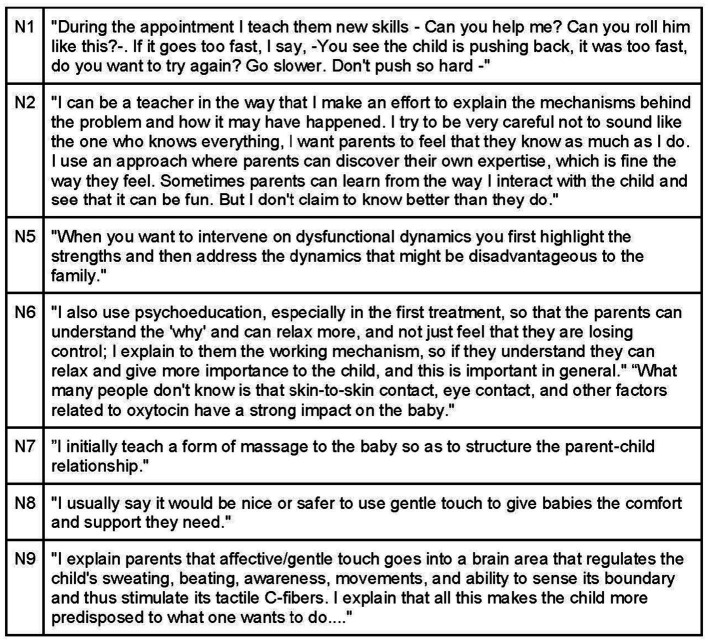
How to help caregivers deal with their child’s needs.

In cases where approaching the child is challenging or when the parent’s state strongly influences the symptoms, treating parents and children together or suggesting separate treatment sessions may be beneficial. Encouraging parents to slow down their breathing and connect with their own body signals can aid in understanding the child’s cues. Osteopaths can involve parents in their child’s health process, highlighting their parenting skills and teaching them reproducible techniques for home use, such as gentle touch and skin-to-skin contact (Figures 17, 18). This approach facilitates a holistic adjustment for both parent and child.

## 4. Discussion

This study explores the contextual factors of osteopathic pediatric care, emphasizing the therapeutic ability, intersubjectivity, and interactions among osteopaths, children, and parents. The child’s development and engagement with the world are deeply intertwined with their relationship and attachment to their caregivers. The triad of osteopath-caregiver-child creates a therapeutic ecological niche that influences the outcomes of care.

**Figure 19:**
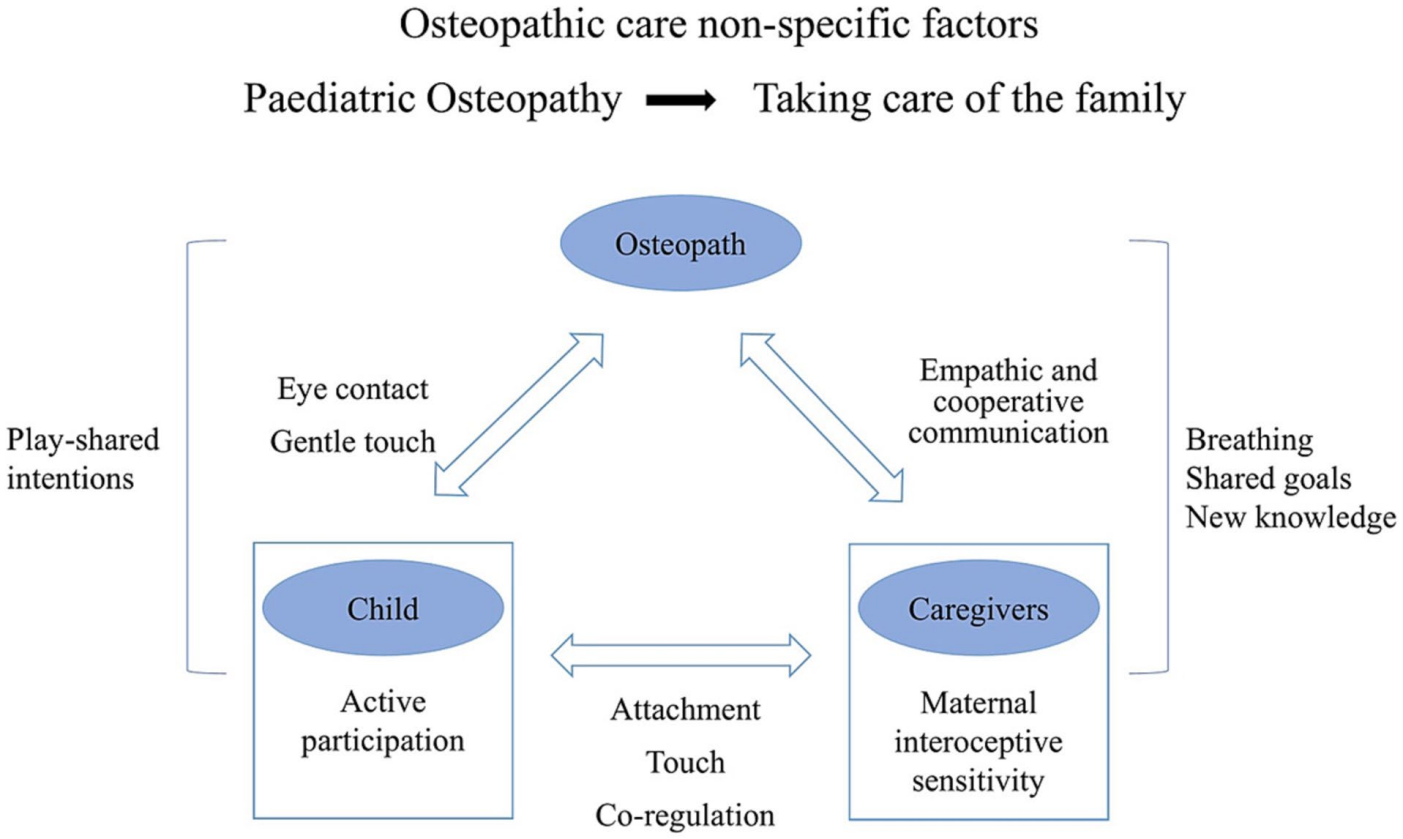
The osteopath-child-caregiver triadic alliance.

**Figure 20:**
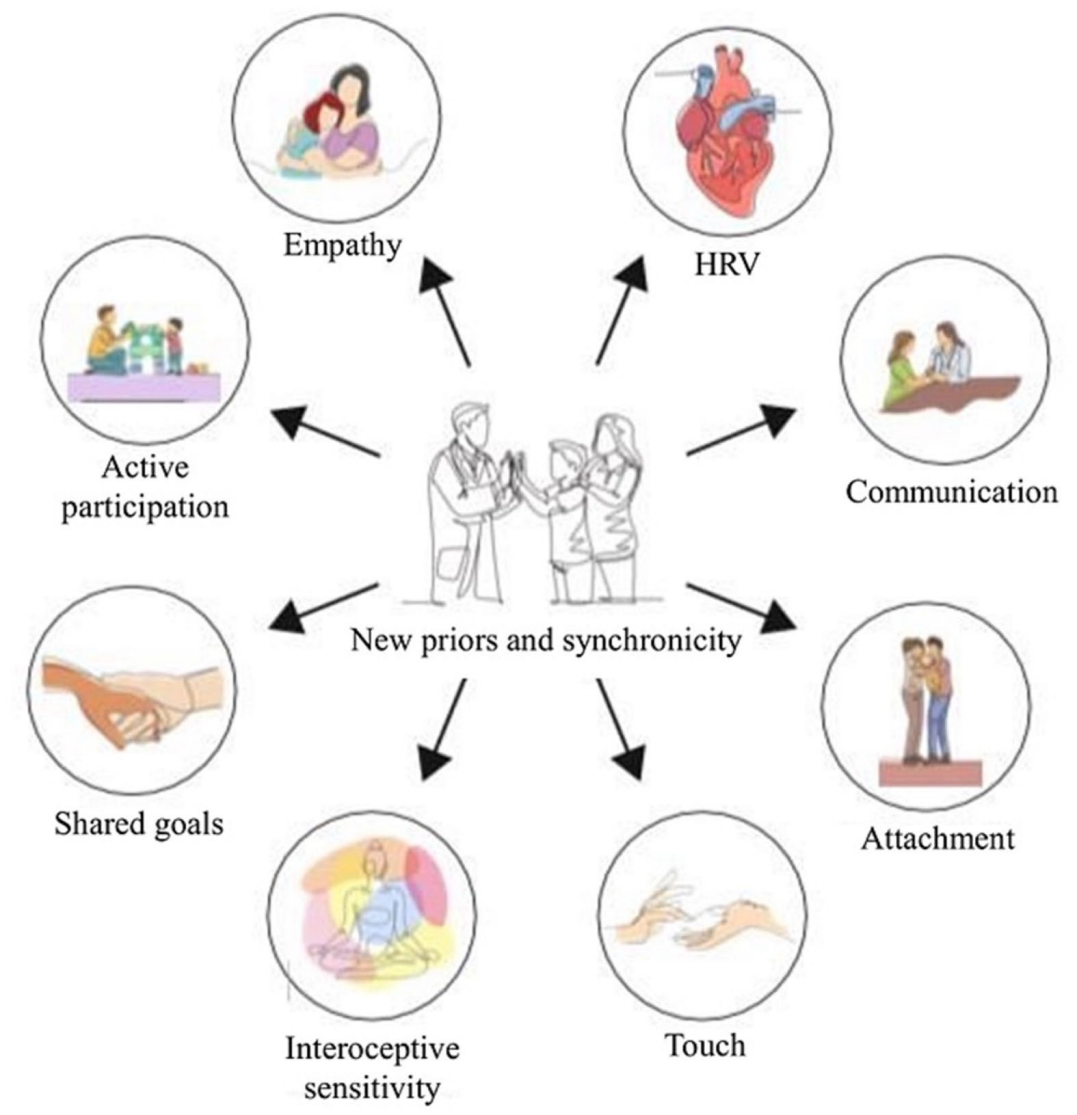
New priors and synchronicity within the triad.

Interviewees emphasize the role of embodied mind in actions, interactions, meaning-making, and sense-making during the therapeutic encounter, shaping the creation of a positive therapeutic ecological niche. Therapeutic interventions, guided by an enactivist perspective, facilitate insight into patients’ feelings and dominant patterns of sense-making (Esteves et al., 2020). The clinical encounter offers opportunities to identify and modify inflexible or ineffective interaction patterns, enabling participatory sense-making (Esteves et al., 2020). While osteopathic care employs both “hands-on” and “hands-off” approaches, our research specifically examines the “hands-off” factors in pediatric osteopathic care. Figures 19, 20 provide an overview of this study’s conceptual framework.

### 4.1. Theme 1 – create a safe environment for parents and children

The interaction between organisms and their environment maintains physiological balance and facilitates adaptation (Ciaunica et al., 2021a,b). The concept of a “Markov blanket” has emerged as a potential framework to understand the system-environment boundary (Kirchhoff et al., 2018; Palacios et al., 2020; Ciaunica et al., 2023). This boundary not only separates the system from its environment but also establishes an intrinsic relationship that connects the organism to its niche (Sims, 2021). Peripersonal space, the area surrounding the body, enables bodily self-awareness, actions, and protection during interactions with the external world (Rabellino et al., 2020).

The environment in which osteopathic visits occur is crucial, as supported by the enactivist interpretation of the therapeutic process (Kim et al., 2022). According to the enactive model, the body and brain are attuned to the interactive environment, shaping one’s sense of self, meaning from past experiences, and predictions about the future (Stilwell and Harman, 2019). The patient’s (child’s) environment is influenced by the physical space and the individuals they interact with, such as parents and caregivers. This reciprocal relationship between the organism and its environment generates a world of meanings based on the organism’s goals, needs, and capabilities (Stendera, 2015).

The interviewed osteopaths actively organize the room to create a child-friendly environment that encourages exploration and active engagement during the visit. They provide toys and furnish the space with soft materials, allowing the child to experience the tactile qualities of the objects and simultaneously learn about their own body.

In the context of the therapeutic process, embodied and embedded systems participate through their presence in the world and mutual connections. This participatory sense-making, where interactions between two individuals generate sense or meaning that goes beyond what either individual could produce alone, is a fundamental aspect (Stilwell and Harman, 2019). It is important for the practitioner to be aware of their own embodied being, as it influences how they perceive themselves and contributes to the co-creation of optimal changes in the child’s function, movement, and participation (Sørvoll et al., 2022).

The establishment of a safe and secure therapeutic environment is crucial for achieving effective therapeutic outcomes. Patients need to feel safe in order to fully engage in the therapeutic process. The development of a healthy therapeutic relationship relies on the therapist’s ability to be fully present and engaged (Geller and Greenberg, 2012). Geller and Porges (2014) emphasized that this relationship requires the therapist to exhibit qualities such as being grounded, focused, stable, open, and receptive to the patient’s experience. By maintaining constant presence, osteopaths can assist patients in regulating their stress response.

Furthermore, respondents highlighted the importance of creating a climate of safety and trust through multidisciplinary collaboration, which aims to provide comprehensive care to both parents and children. This approach aligns with existing literature (Vaughan et al., 2017; Lunghi et al., 2021) and the World Health Organization’s promotion of “integrated health care.” Effective collaboration with other health professionals necessitates expertise in the pediatric field and the availability of scientific evidence to support the osteopathic approach (Cerritelli et al., 2021a).

### 4.2. Theme 2 – communication

The therapeutic alliance serves as a cooperative relationship between the clinician and the patient, playing a vital role in person-centered care. It fosters trust, recognition, and alignment with an individual’s unique needs through cooperative communication (Miciak et al., 2019; Ryu et al., 2021; Kim et al., 2022). In the context of osteopathic care, it is essential to go beyond mere knowledge and execution of techniques. It also relies on the practitioner’s ability to perceive the intended meaning behind the child’s gestures, postures, movements, and expressions, as well as the timing and emotional attunement during reciprocal embodied communication (Sørvoll et al., 2022).

In clinical encounters involving caregivers, children, and parents, individuals’ lived bodies are mutually coupled through eye contact, gaze, facial expressions, voice, gestures, positioning, movements, and intentional actions (Sørvoll et al., 2022). Therapeutic interactions are bidirectional and interactive, emphasizing the importance of therapeutic presence in enhancing the patient’s sense of safety (Fuchs and De Jaegher, 2009; Liem, 2019).

#### 4.2.1. Communication with parents

Interlocutors strive to align their mental states for cooperative communication (Vasil et al., 2020). In therapy, treatment goals are discussed, leading to a transparent and stable therapeutic relationship (Liem, 2019). Both patient and therapist acknowledge patient and parental needs, including the desire for control and coping strategies (Liem and Heede, 2016). Empathy involves activating personal representations to understand others’ states and relies on interoceptive awareness, with empathy and self-awareness linked to the anterior insular cortex (Fan et al., 2011; De Waal and Preston, 2017; López-Solà et al., 2019; Schaefer et al., 2021). Empathic tendencies contribute to robust therapeutic alliances and positive treatment outcomes, fostering brain–brain synchrony (Goldstein et al., 2018).

#### 4.2.2. Communication with children

The child’s body engages in continuous interaction with the environment, fostering participatory sense-making in clinical encounters with osteopaths (Fuchs and De Jaegher, 2009). Upon experiencing something new, such as visiting the osteopath’s office, children naturally engage in physical exploration, which aligns with the goal of inviting them to participate in spontaneous, shared exploration (Ciaunica and Crucianelli, 2019). Research shows that children derive satisfaction from shared intentions, as it activates the reward system and reinforces trust, empathy, and the therapeutic relationship (Green and Hollander, 2010; Pauli et al., 2016; Feldman, 2017).

Respondents noted the combination of touch and active visual stimulation, such as gaze and eye contact, which elicits muscle and hormonal responses in the child’s body, generating interoceptive sensations linked to past experiences and guiding ongoing responses (Barrett and Bar, 2009). Unlike other senses, touch encompasses the entire body surface, developing alongside the nervous system during gestation (Montirosso and McGlone, 2020). The ability of osteopaths to utilize affective touch, mediated by CT fibers, in initial interactions activates these fibers, leading to autonomic, neurochemical, and behavioral responses that may enhance the child’s receptivity to osteopathic manipulative treatment (Pawling et al., 2017; Cerritelli et al., 2021b). This process of participatory sense-making fosters a “we-space” in which the practitioner and child synchronize bodily actions and interactions, co-creating meaning and coordination (Sørvoll et al., 2022).

### 4.3. Theme 3: look for synchrony and attachment

The osteopath’s role in interacting with the child and their parents involves observing the family dynamics and relationships that shape the child’s development. These social encounters between the child and their interlocutor rely on the exchange of lived bodily experiences, where perception and mutual influence occur simultaneously. Skillful observation allows the osteopath to gain insights into the communication and mutual influence between the parent and child.

The processing of bodily information begins during the early stages of life, possibly even during prenatal development (Zieber et al., 2015). The infant’s interaction with the mother’s body, mediated by CT fibers, precedes their engagement with the mother’s mind (Ciaunica et al., 2021a). The fetus establishes meaning and is influenced by its interaction with the uterine environment, thereby beginning to embody and situate itself within its surroundings (Quintero and De Jaegher, 2020).

Within the first year of life, the infant experiences implicit synchrony between their bodily activities and those of the mother, as evidenced by EEG activity, heart rate, and neuroendocrine responses (Van Puyvelde et al., 2019). Research on the mu rhythm suggests that infants possess a mirror mechanism enabling them to establish sensorimotor correspondences between their own body parts and those of others (Meltzoff et al., 2018). From birth, the mother and infant engage in embodied interactions, encompassing routines such as diaper changing, feeding, and playful exchanges. Through these multimodal reciprocal exchanges, mother and infant coordinate their behaviors and bodies, contributing to rhythmic cycles of co-regulation, often referred to as “synchrony,” “mutual regulation,” or “attunement” (DiCorcia and Tronick, 2011).

Synchrony encompasses the interpersonal coordination of behavioral and neurophysiological rhythms, facilitating sensory processing, learning, emotion and arousal regulation, self-regulation, and the establishment of communicative relationships (Beebe et al., 2018; Wass et al., 2020; Carozza and Leong, 2021). Caregivers achieve biobehavioral synchrony when responding to a helping stimulus by activating various subcortical regions, including the hypothalamus, amygdala, and dopaminergic reward circuitry (Carozza and Leong, 2021). Montirosso and McGlone (2020) observed that mother–child dyads achieve biobehavioral synchrony through maternal touch and interoceptive sensitivity.

#### 4.3.1. Touch

The role of affective touch in fostering interpersonal body attunement and supporting the infant’s bodily self-representation is a subject of hypothesis (Bremner and Spence, 2017). Tactile behaviors, such as skin-to-skin contact and gentle touch, employed by the mother during the early stages of the infant’s life, have a profound impact. These tactile stimulations positively influence various aspects, including sleep patterns, regulation of temperature and heart rate, cognitive development, and the quality of attachment. Conversely, the absence of tactile stimulation has been observed to have negative consequences on the infant’s response to stress (McLaughlin et al., 2015). Therapeutic touch has the potential to elicit an affective response (Geri et al., 2019) and influence the body schema/perception (Viceconti et al., 2020).

The close contact between mother and infant during the initial months of life allows for the mutual regulation of each other’s physiological activities, creating a form of physiological body-to-body synchrony known as body tuning or co-regulation (Normann et al., 2015; Atzil and Gendron, 2017; Kim et al., 2022). Through this co-regulation, the mother and infant engage in a reciprocal process of influencing and aligning their physiological states. This synchronization contributes to the establishment of a strong bond between them and promotes healthy development.

#### 4.3.2. Interoceptive sensitivity

Mothers need awareness of their own bodily experiences to interpret and respond to their child’s body signals (Montirosso and McGlone, 2020). Maternal interoceptive sensitivity, striking a balance between self-awareness and awareness of the infant, regulates bodily sensations to support the infant’s regulatory abilities and restore physiological co-regulation (Abraham et al., 2019).

The Reciprocal Regulation Model by Tronick (2004) explains how mothers and infants alternate between attuned and asynchronous states, using “repair” to recover from asynchronies. Infants experiencing more frequent dyadic repairs show increased vagal reactivity, reflecting adaptive parasympathetic functioning and effective stress regulation (Provenzi et al., 2015).

In summary, maternal interoceptive sensitivity and the repair process in mother-infant interactions contribute to regulating physiological states, fostering attunement, and enhancing the infant’s stress coping abilities.

### 4.4. Theme 4 – synchronizing

When discrepancies arise between parent and child, subsequent repairs that restore synchrony are crucial for building trust, promoting development, and fostering a sense of participation and action in infants (Fuchs and De Jaegher, 2009).

Based on the aforementioned findings, we propose that osteopaths can play a role in enhancing the success of parent–child interactions by providing skills, tools (such as touch and skin-to-skin contact), and facilitating a heightened perception of self and the relationship. By supporting the sense of participation and action in both the child and parent, the osteopath contributes to the reconstruction of generative models and alignment of predictive models, replacing erroneous or dysfunctional beliefs with new meanings through participatory sense-making and achieving interpersonal synchrony (McParlin et al., 2022).

Each individual’s mind-brain is an active agent that interacts with the environment and is shaped through the sense of participation and action, generating meanings and forming the sense of self (Montirosso and McGlone, 2020). In the osteopathic clinical encounter, establishing synchronicity is vital, as it provides a foundation for shared narratives and trust, which are necessary for dispelling erroneous beliefs. Aligning mental states to achieve biobehavioral synchrony between the patient and practitioner allows for the regulation of allostasis, restoration of homeostasis, and effective management of the clinical problem (McParlin et al., 2022).

By facilitating eye and touch contact between parents and their child during treatment, osteopaths support the child’s allostatic regulation and foster secure attachment (McGlone et al., 2017). Dynamic stroking, as demonstrated by Manzotti et al. (2019), activates CT fibers and the posterior insular cortex in both mothers and infants, promoting the development of regulation, synchronization, and anticipation of sensory stimuli. Embodied repair, such as breastfeeding and skin-to-skin contact, allows the infant to reduce discrepancies within the interoceptive system by utilizing the mother’s body (Sørvoll et al., 2022). Therefore, involving parents in the infant’s management or treating the infant together with the parents can enhance secure attachment, amplifying the parasympathetic response to affective touch, similar to how osteopathic care promotes autonomic balance through preganglionic cholinergic action (Cerritelli et al., 2020).

### 4.5. Limitations

The selection of participants was facilitated by a teacher and researcher, potentially leading to a polarization of perspectives and introducing a potential selection bias. Additionally, although the participants were sourced from four different countries, they may not adequately represent the entire osteopathic population, and the representation across countries is not well-balanced.

Throughout the online meetings, there were instances of connection loss, which posed challenges in achieving mutual understanding and potentially resulted in misinterpretations. However, to address this concern, a member checking strategy was implemented to validate the collected data.

Furthermore, the interpretation of the interviews may have been influenced by self-confirmation biases, as the author possessed pre-existing opinions on certain specific topics. To mitigate this issue, a double-checking process and coding by another qualitative researcher were employed.

## 5. Conclusion

This article explores the contextual factors that contribute to effective osteopathic pediatric care. It highlights the importance of creating a safe and secure therapeutic environment, where both parents and children feel comfortable and supported. By being fully present and engaged, osteopaths can establish a strong therapeutic alliance and foster trust, recognition, and alignment with individual needs.

Effective communication is a key aspect of osteopathic care, and it is crucial to establish cooperative and empathetic communication with both parents and children. Osteopaths should be attuned to the child’s nonverbal cues, such as gestures, postures, movements, and expressions, and engage in reciprocal embodied communication. This level of communication allows for a deeper understanding of the child’s needs and facilitates the therapeutic process.

The study emphasizes the significance of synchrony and attachment in osteopathic care. By observing family dynamics and relationships, osteopaths can gain insights into the communication and mutual influence between parents and children. Biobehavioral synchrony, which involves the coordination of behavioral and neurophysiological rhythms, plays a vital role in sensory processing, learning, emotion and arousal regulation, self-regulation, and the establishment of communicative relationships. Touch and interoceptive sensitivity are essential in fostering synchrony and attachment, enabling physiological co-regulation and the development of a strong bond between parents and children.

Overall, osteopaths can enhance parent–child interactions by providing skills, tools, and facilitating heightened self-perception and relational awareness. By supporting the sense of participation and action in both the child and parent, osteopaths may contribute to the reconstruction of generative models and alignment of predictive models. This promotes participatory sense-making and achieves interpersonal synchrony, which is crucial for building trust, dispelling erroneous beliefs, and fostering secure attachment between parents and children.

In conclusion, this research illuminates the multifaceted dimensions inherent in osteopathic pediatric care, highlighting the pivotal role of the therapeutic environment, effective communication, synchrony, and attachment. These elements collectively contribute to the comprehensive and holistic care provided by osteopaths to parents and children. Beyond the realm of osteopathy, these findings hold implications that resonate with other health professions where touch serves as a vital conduit for communication and care, such as physiotherapy and nursing.

As this study elucidates the intricate interplay of contextual factors, it prompts osteopaths to recognize their potential in optimizing therapeutic outcomes. By nurturing an environment that encapsulates safety, security, and connection, practitioners can foster not only physical healing but also emotional well-being for both parents and children. Moving forward, there is a compelling avenue for further investigation into the enduring impact of creating such a nurturing therapeutic milieu on the overall development and well-being of children undergoing osteopathic pediatric care.

In addition to the qualitative methodologies employed in this study, future research could incorporate quantitative approaches to provide further empirical support for these insights. The expansion of research endeavors to encompass a broader range of countries would amplify the diversity of perspectives and enrich the generalizability of the findings.

Ultimately, this research transcends the boundaries of osteopathy, resonating across healthcare disciplines where touch serves as a pivotal vehicle for effective communication, healing, and nurturing care.

## Data availability statement

The raw data supporting the conclusions of this article will be made available by the authors, without undue reservation.

## Ethics statement

The studies involving humans were approved by Malta ICOM Educational Ethics Commitee. The studies were conducted in accordance with the local legislation and institutional requirements. The participants provided their written informed consent to participate in this study.

## Author contributions

CA: Conceptualization, Data curation, Formal analysis, Investigation, Methodology, Project administration, Writing – original draft, Writing – review & editing. FC: Conceptualization, Investigation, Methodology, Supervision, Validation, Writing – review & editing. LB: Formal analysis, Investigation, Writing – review & editing. JE: Conceptualization, Formal analysis, Funding acquisition, Investigation, Methodology, Supervision, Writing – review & editing.
